# The association of the MTHFR C677T polymorphism with inflammatory bowel diseases in the Israeli Jewish population: An example of genetic heterogeneity

**DOI:** 10.1097/MD.0000000000005611

**Published:** 2016-12-23

**Authors:** Amir Karban, Tzah Feldman, Matti Waterman, Ronit Leiba, Edna Efrati

**Affiliations:** a Department of Internal Medicine C, Rambam Health Care Campus; b Bruce Rappaport Faculty of Medicine, Technion-Israel Institute of Technology; c Laboratory of Toxicology, Pharmacology and Pharmacogenetics, Israel Poison Information Center, Rambam Health Care Campus; d Department of Gastroenterology, Rambam Health Care Campus; e Epidemiologic Unit, Rambam Health Care Campus, Haifa, Israel.

**Keywords:** Ashkenazi Jews, Crohn's disease, inflammatory bowel diseases, MTHFR gene, ulcerative colitis

## Abstract

MTHFR C677T is a common gene polymorphism that has been shown to be associated with hyperhomocysteinemia. Studies on the role of MTHFR in inflammatory bowel diseases (IBD) have yielded conflicting results, perhaps due in part to genetic heterogeneity. The prevalence of the MTHFR C677T variant allele varies according to Jewish subpopulations: Ashkenazi vs non-Ashkenazi. The aim of this study was to examine the association between MTHFR C677T genotype and IBD in the different Jewish populations.

DNA samples were assessed for the presence of the MTHFR C677T variant allele in 445 Jewish Israeli IBD patients: 338 with Crohn's disease [CD] (214 Ashkenazi and 124 non-Ashkenazi Jews) and 107 with ulcerative colitis [UC] (73 Ashkenazi and 34 non-Ashkenazi Jews), and in 347 healthy controls: 173 Ashkenazi and 174 Non-Ashkenazi Jews. Possible genotype–phenotype associations were investigated.

We showed a significantly higher frequency of MTHFR 677T variant genotypes in non-Ashkenazi CD patients: Odds ratio of 1.86 for heterozygotes (CT) and 2.89 for homozygotes (TT) compared to non-Ashkenazi healthy controls. No significant association was found for UC in non-Ashkenazi patients or for CD or UC in Ashkenazi patients.

Our findings suggest that the MTHFR 677T variant may contribute to the risk of CD in non-Ashkenazi but not Ashkenazi Jews. This may result from genetic heterogeneity and highlights the complexity of the genetic etiology of IBD.

## 1 Introduction

5,10-Methylenetetrahydrofolate reductase (MTHFR) plays a major role in the metabolism of homocysteine and folate, and may thus modulate homocysteine levels. A common thermolabile variant of MTHFR was characterized and found to correspond to the MTHFR C677T gene polymorphism (rs1801133). This polymorphism is associated with hyperhomocysteinemia, as well as with various cancers,^[1]^ cardiovascular diseases,^[2]^ and thromboembolism.^[3]^ Increased levels of homocysteine and increased risk of thromboembolism have been described in inflammatory bowel diseases (IBD).^[4,5]^ Although the MTHFR gene is located on chromosome 1 (1p36.3), a locus found to be associated with IBD risk,^[6,7]^ other genome-wide association studies (GWAS) did not identify the MTHFR C677T polymorphism in individuals with IBD.

Association studies examining MTHFR polymorphisms and IBD show conflicting results. The first published study in 1999^[8]^ showed an association of MTHFR C677T to IBD (Odds ratio (OR) 2.64, 95% confidence interval (CI) 1.39–5.08). A meta-analysis, including 13 studies from populations of different ethnic descent, altogether 1849 and 2249 IBD cases and controls for MTHFR C677T, respectively, found no statistically significant association between the MTHFR C677T polymorphism and the risk of Crohn's disease (CD) or Ulcerative colitis (UC).^[9]^ Failure of the meta-analysis to corroborate the association between IBD and MTHFR C677T may be due to the heterogeneity of the populations studied. Indeed, heterogeneity in the risk of IBD-associated loci among different populations has been described for several important IBD loci. For example, NOD2/CARD15 (nucleotide-binding oligomerization domain-containing protein 2) gene polymorphisms are associated with CD; however, the 3 risk alleles R702W, G908R, and 1007fsInsC in NOD2 that are associated with susceptibility to CD, have demonstrated remarkable heterogeneity across ethnicities and populations, with regional variation even across Europe. In the Japanese, Korean, Chinese and Indian populations, the NOD2/CARD15 variants are not associated with CD.^[10]^

The Jewish population in Israel is heterogeneous and is historically divided into 2 major groups: Ashkenazi Jews from Central and Eastern Europe and non-Ashkenazi Jews from the Mediterranean, North Africa, and Asia. The incidence and prevalence of inflammatory bowel diseases has been reported to be 2- to 4-fold higher in Ashkenazi Jews than in non-Jewish Caucasians.^[11]^

We previously showed that the carrier rate of the 3 *NOD2* polymorphisms R702W, G908R, and 1007fsInsC is higher in Ashkenazi Jewish CD patients than in non-Ashkenazi Jews (47.4% vs 27.5%, *P* = 0.034).^[12]^ We also showed that Ashkenazi Jews display a 1.9-fold higher frequency of variant MTHFR 677T than do non-Ashkenazi Jews (*P* < 0.001).^[13]^ However, no study to date has focused on the association of the MTHFR C677T polymorphism with IBD in the Jewish population. The aim of the present study was to examine the association of the MTHFR C677T polymorphism with IBD in Ashkenazi versus non-Ashkenazi Jews in order to elucidate controversial findings of associations between the MTHFR C677T polymorphism and IBD. We assumed that an association between MTHFR C677T polymorphism and IBD can be found in the heterogeneous Jewish population in Israel.

## 2 Materials and methods

### 2.1 Study design

In order to examine the association of the MTHFR C677T polymorphism with IBD in the Jewish population, DNA samples were assessed for the presence of the MTHFR C677T variant in Jewish Israeli individuals with IBD and healthy Jewish controls. The subjects and the controls were divided to 2 subgroups by ethnicity (Ashkenazi and non-Ashkenazi Jews). Several comparisons were conducted between subjects and controls genotype frequencies according to ethnicity.

### 2.2 Study subjects

Starting from 1996, we recruited 445 Jewish Israeli individuals with IBD (338 with CD and 107 with UC) from Rambam Medical Center in Haifa, Israel. The diagnosis of UC and CD was based on established criteria. An appropriately trained gastroenterologist confirmed all diagnoses. Confirmation required first-hand review of endoscopic, pathology and radiology reports, and operative notes.

### 2.3 Healthy controls

We used for comparison, DNA samples of 347 healthy Jewish Israeli controls, 173 Ashkenazi Jews, and 174 non-Ashkenazi Jews, that were collected by the National Laboratory for the Genetics of Israeli Populations, Department of Human Molecular Genetics & Biochemistry, Sackler Faculty of Medicine, Tel-Aviv University.^[13]^

### 2.4 Phenotypic evaluation

Age at diagnosis, tobacco use, ethnicity, and phenotypic parameters (extent of disease, perianal disease in CD, extra-intestinal manifestations) were determined from medical records, questionnaires, and interviews. Ashkenazi and non-Ashkenazi ethnicities were carefully assigned on the basis of the birthplace of the 4 grandparents. Non-Ashkenazi ethnicity includes all individuals who had at least 3 Sephardic grandparents (originating from Spain, Portugal, Iraq, and North Africa). Georgian, Indian, and Ethiopian Jews were not included.

Patients were considered smokers if they smoked a minimum of 7 cigarettes per week for at least 1-year anytime during their life. Family history of IBD was the occurrence of IBD in a first cousin or more closely related relative.

### 2.5 DNA samples

Genomic DNA was extracted from peripheral blood samples using the Qiamp DNA mini kit (Qiagen, Germany) according to the manufacturer's protocol.

### 2.6 MTHFR genotyping

DNA samples were assessed for the presence of polymorphism C677T in *MTHFR* using the PCR followed by probe-free high-resolution melting technology (HRM), a genotyping technique based on the effect of DNA changes on amplicon melting.

DNA samples were amplified using HRM-enabled real-time PCR, Rotor-Gene 6000 (Corbett Research, UK) with Fast Plus EvaGreen qPCR master mix (Biotium, USA) and 2 primers: 5′-CTTTGAGGCTGACCTGAAGC-3′ and 5′-AGAAAAGCTGCGTGATGATGA-3′.

High-resolution melting technology (HRM) is a genotyping technique that incorporates an intercalating dye into a double-stranded PCR amplicon. The dye fluoresces brightly when it is bound to double-stranded amplicon. Thus, incrementing the temperature of an amplicon to melting point while measuring the fluorescence of the dye results in the generation of a sequence specific melting curve. Normalization and comparison between different shapes of melting curves allows sensitive discrimination between different genotypes and identification of heterozygotes, homozygotes, and wild type variants.

### 2.7 NOD2ICARD15 mutation analysis

Patients were genotyped for the following 3 gene polymorphisms in *NOD2*: Arg702Trp, Gly908Arg, and Leu1007finsC using the restriction enzyme digestion assay as described elsewhere.^[14]^

### 2.8 Statistical analysis

Statistical analysis was performed using SPSS version 21.

Individuals with any missing data were excluded from the statistical analysis regarding the data that were missing. However, they were included in other analyses regarding their available data.

The Fisher exact test and the chi square test were used to analyze differences between groups (example: CD Ashkenazi patients and CD non-Ashkenazi patients), in the categorical parameters (gender, smoking, family history of IBD, disease extent, CD-type, CD-perianal, extra-intestinal manifestations, and NOD2 carrier). T-test analysis was used to analyze data according to differences in patient age (a continuous parameter).

Odds ratio with 95% confidence interval for *MTHFR* C677T was calculated for measuring the strength of the association for Jewish healthy controls (Ashkenazi vs non-Ashkenazi) and for IBD patients versus healthy controls (among Ashkenazi and non-Ashkenazi Jews).

Adjustment for multiple comparisons was made by the Bonferroni method.

*P* < 0.05 was considered as significant.

### 2.9 Ethical considerations

Informed consent for participation in molecular genetic studies was obtained from all study subjects and ethical approval from the local Helsinki Committee.

## 3 Results

All recruited individuals were included in the study. Individuals with any missing data were excluded from the statistical analysis regarding the data that were missing. However, they were included in other analyses regarding their available data.

The study included 338 Jewish CD patients, (Table 1): 214 Ashkenazi and 124 non-Ashkenazi. In total, 53% of the patients were males and the mean age at diagnosis was 24.8 ± 13 years. Phenotypic characteristics were similar in Ashkenazi and non-Ashkenazi patients except for a higher prevalence of smokers among non-Ashkenazi patients, 31% versus 18%, *P* = 0.009; a slightly higher proportion with family history of IBD, 31% versus 21%; and a significantly lower carrier rate of NOD2 mutations, 24% versus 41%.

**Table 1:**
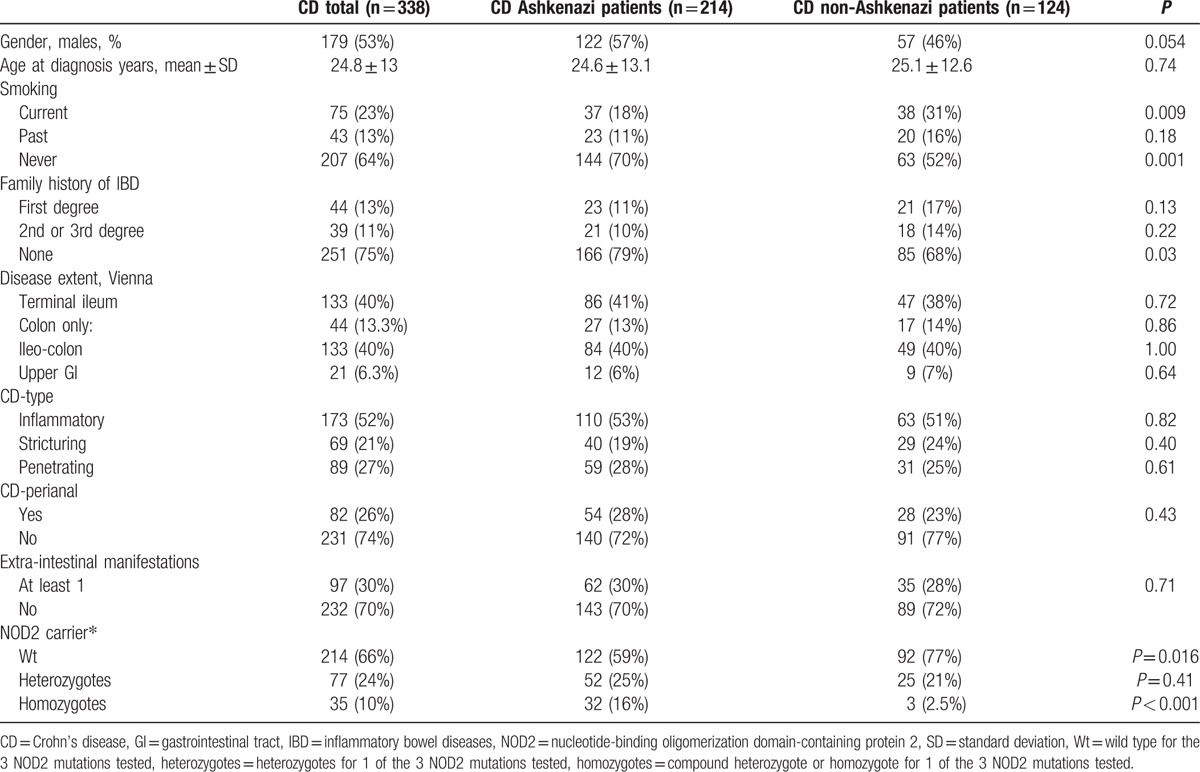
Crohn's disease patients cohort characteristics.

The study included 107 UC patients (Table 2): 73 of Ashkenazi and 34 of non-Ashkenazi descent; 54% of the patients were males; the mean age at diagnosis was 30.7 ± 15.1 years. Phenotypic characteristics were similar in Ashkenazi and non-Ashkenazi patients.

**Table 2:**
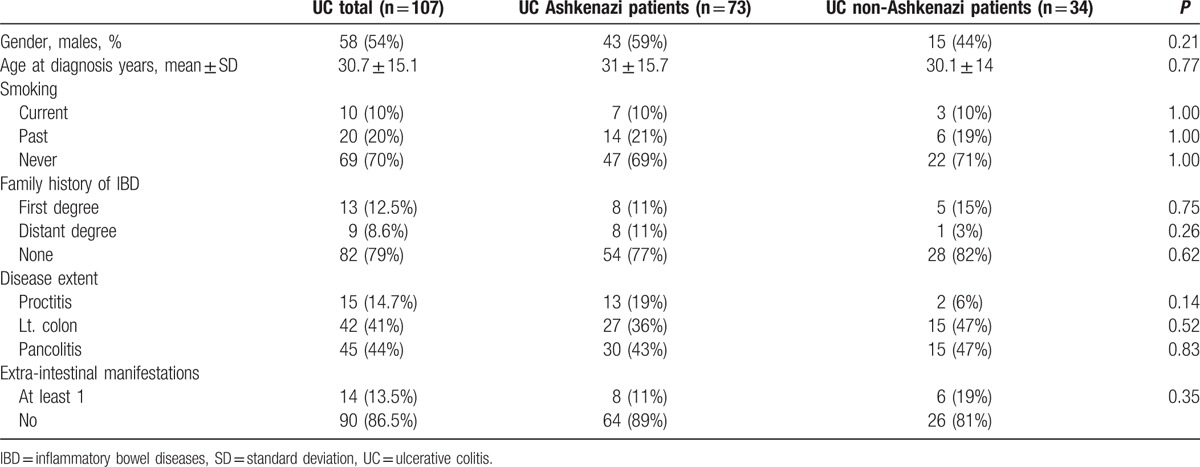
Ulcerative colitis patients cohort characteristics.

Comparing Ashkenazi with non-Ashkenazi Jews in the control groups, we found that the frequency of the MTHFR 677T variant genotype was significantly higher in Ashkenazi than non-Ashkenazi Jews (44.5% vs 21.3%, OR 2.96, 95% CI 2.12–4.14, *P* < 0.0001) (Table 3). Because of the difference in the genotype frequencies between control Ashkenazi and non-Ashkenazi Jews, the following comparisons were conducted according to ethnicity.

**Table 3:**
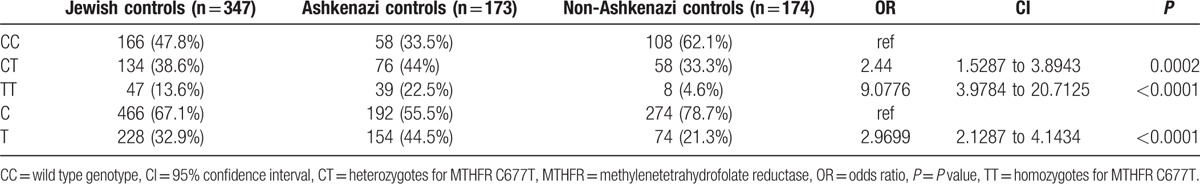
MTHFR C677T frequency in Jewish healthy controls.

**Figure 1:**
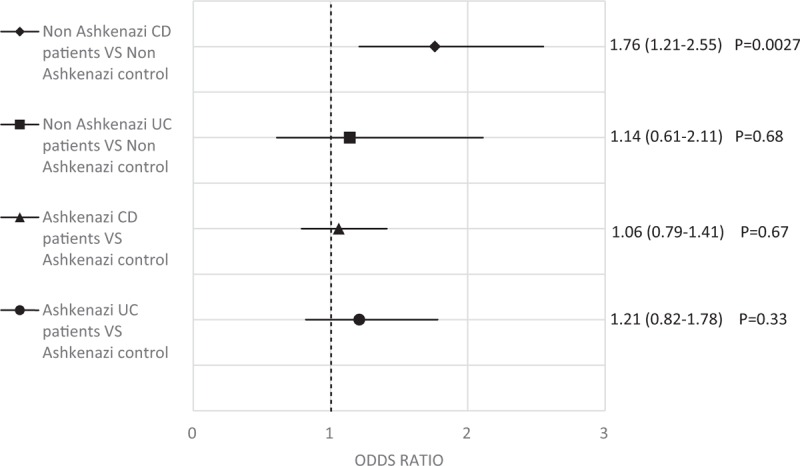
Odds ratio with 95% confidence interval of several MTHFR T allele comparisons conducted in this study. Each shape represents different comparisons between IBD patients and ethnically matched healthy controls, as mentioned on the left side of the graph. On the right side Odds ratio, 95% confidence interval and *P* values for each comparison are mentioned. *P* < 0.05 was considered as significant. T allele prevalence is significantly higher among non-Ashkenazi CD patients compared to non-Ashkenazi controls. CD = Crohn's disease, IBD = inflammatory bowel diseases, MTHFR = methylenetetrahydrofolate reductase, UC = ulcerative colitis.

Among Ashkenazi Jewish individuals, no significant difference was found in MTHFR C677T allele frequencies, between those with and without CD, or with and without UC (Fig. 1).

Among non-Ashkenazi individuals, 45% of those with CD are heterozygotes (CT) for MTHFR polymorphism, compared to 33% of those without CD (OR 1.86, 95% CI 1.14–3.03, *P*-value = 0.0127). However, 10% of those with CD are homozygotes (TT) for MTHFR polymorphism, compared to 4.6% of those without CD (OR 2.89, 95% CI 1.11–7.48, *P*-value = 0.0286). No significant difference was found in the frequency of heterozygotes or homozygotes, between those with and without UC.

A total of 32% of the alleles of those with CD are T allele of MTHFR, compared to 21% for those without CD (OR 1.76, 95% CI 1.21–2.55, *P*-value = 0.0027) (Fig. 1). No significant association was found in the frequency of T allele, between those with and without UC.

Further phenotype-genotype correlations were conducted in non-Ashkenazi CD patients (Table 4). No MTHFR C677T phenotype–genotype correlations were found in this subgroup of patients. No interaction (epistasis) was found between NOD2 genotypes and MTHFR C677T genotypes in this population as well.

**Table 4:**
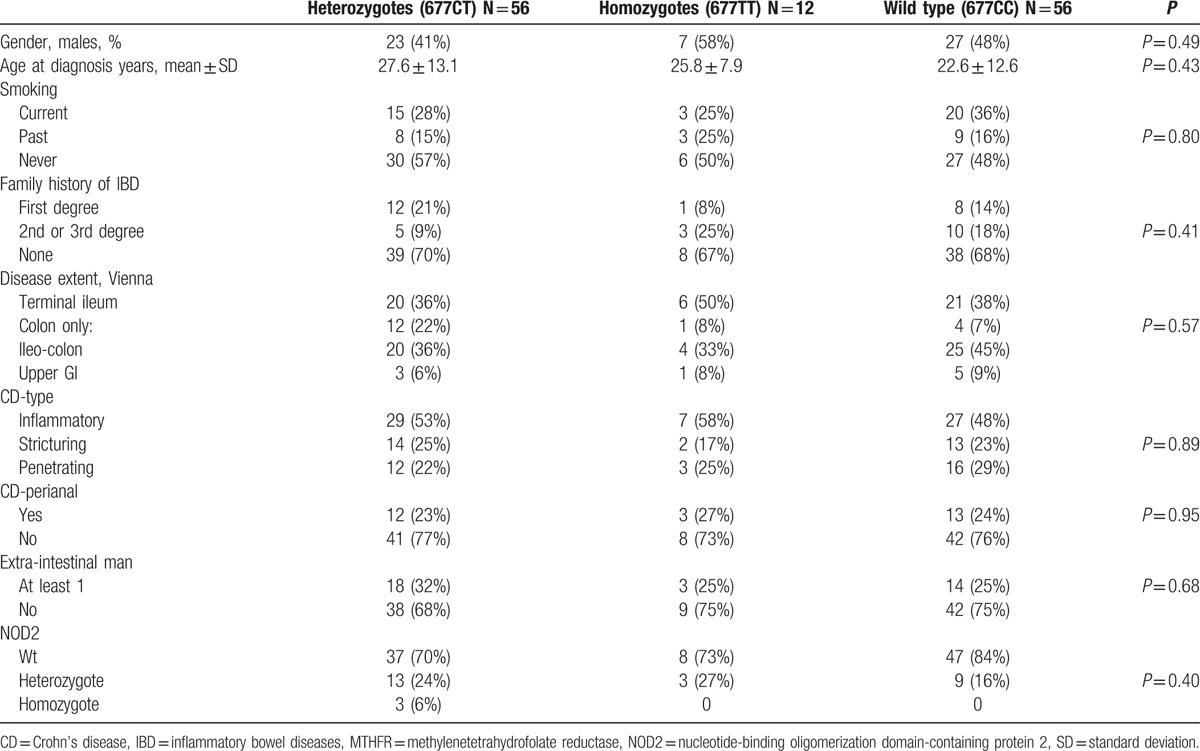
MTHFR C677T phenotype–genotype correlations of in non-Ashkenazi Crohn's disease patients (n = 124).

## 4 Discussion

This study compared frequencies of the MTHFR 677T variant allele in 445 Jewish Israeli individuals with IBD (338 with CD and 107 with UC) to 347 Jewish healthy controls. No study to date has focused on the association of the MTHFR C677T polymorphism with IBD in the Jewish population. We showed a significantly higher frequency of MTHFR 677T variant in non-Ashkenazi CD patients: odds ratio of 1.86 for heterozygotes (CT) and 2.89 for homozygotes (TT), compared to 174 non-Ashkenazi healthy controls. We did not find any correlation between MTHFR C677T genotype and age at diagnosis, smoking status or disease phenotype, or with NOD2 gene polymorphism carrier status among the non-Ashkenazi CD patients.

No significant association was found between the MTHFR C677T polymorphism and UC in non-Ashkenazi Jewish patients, though a small sample size limited the statistical power.

Unlike Ashkenazi Jews who are a definite population isolate,^[15]^ non-Ashkenazi Jews show significant genetic heterogeneity.^[16]^ In broad terms, they could be a mixture of Spanish Moroccan, North-African, Iraqi, Yemenite, Georgian, Indian, Ethiopian Jews, and so on. We included in the study individuals that at least 3 of their grandparents originated from Spain, Portugal North Africa, and Iraq. It has been described in the literature how population substructure can result in false-positive association results.^[17]^ This is particularly liable to be a problem in candidate gene studies in which corrections for genome-wide population differences are not undertaken.

The association found between MTHFR C677T and CD is in agreement with some previous studies. In a Caucasian population in Ireland, 17.5% of UC and 16.8% of CD patients were homozygous for the C677T variant compared with 7.3% of controls.^[8]^ Xu et al^[18]^ reported higher prevalence of the MTHFR C677T polymorphism in Chinese UC compared to healthy patients. However, most studies that compared IBD patients and healthy controls did not find a difference in the frequency of MTHFR C677T variant.^[19–26]^ Some of the studies that showed “negative result” were carried out on populations from Turkey and North Africa. These populations are close in ethnicity to non-Ashkenazi Jews, whose ancestors migrated from Spain and Portugal to North Africa and Turkey during the fifteenth century.

Yasa et al^[19]^ studied a very small cohort of 27 Turkish IBD patients. Heterozygosity of MTHFR C677T polymorphism was found in 10 of 27 (37%) patients with IBD and 15 of 47 (32%) controls (*P* > 0.05). Homozygosity was detected in 4 patients (14.9%) with IBD and 3 (6.3%) controls (*P* > 0.05). Senhaji et al^[20]^ studied Moroccan patients with IBD and concluded that the genetic risk for IBD is not modulated by the MTHFR C677T polymorphism.

It is important to mention the trans-ancestry association study of IBD, with genome-wide or immunochip genotype data from an extended cohort of 86,640 European individuals and immunochip data from 9846 individuals of East Asian, Indian, or Iranian descent. Genetic heterogeneity was observed between divergent populations at several established risk loci. The MTHFR C677T polymorphism was not observed at all, but the Sephardic Jewish population was not represented in this study.^[27]^

A characteristic of IBD is an increased risk of thromboembolic events due to inflammation, nutritional deficiencies, hospitalizations, surgery, and inherited prothrombotic factors.^[28]^ The MTHFR C677T polymorphism has been shown to lead to a 25% increase in homocysteine plasma levels in homozygous carriers. The effect of the MTHFR C677T variant on venous thromboembolism risk varies among studies, and a recent meta-analysis found a weak effect (20% risk increase).^[29]^

The magnitude of association between homocysteine metabolism and IBD remains unknown and also the association between hyperhomocysteinemia and thrombosis remains controversial in IBD. A meta-analysis by Oussalah et al^[30]^ examined these issues. It evaluated 29 studies and concluded that the plasma folate level was inversely correlated with risk of IBD associated with MTHFR C677T polymorphism, indicating a gene–environment interaction. One of the limitations of our study is that it does not consider the possible differences in folate consumption amongst participants. We also did not have reliable data on the occurrence of thromboembolic events and homocysteine levels among our IBD cohort and controls. Therefore, we could not study the association of MTHFR C677T with IBD and thromboembolic events.

In summary, the prevalence of the MTHFR C677T variant genotype in IBD showed discordant results, most likely due to regional and ethnic variations in the prevalence of this polymorphism between the populations studied.

The ALlele FREquency Database (ALFRED), which is a resource of gene frequency data on human populations supported by the U.S. National Science Foundation, summarizes allele frequencies of the MTHFR C677T polymorphism in different populations (Table 5) (http://alfred.med.yale.edu). These data show low frequency of the polymorphic allele C677T in the African population and especially high frequencies in Mexico, Italy,^[31]^ and among Ashkenazi Jews.

**Table 5:**
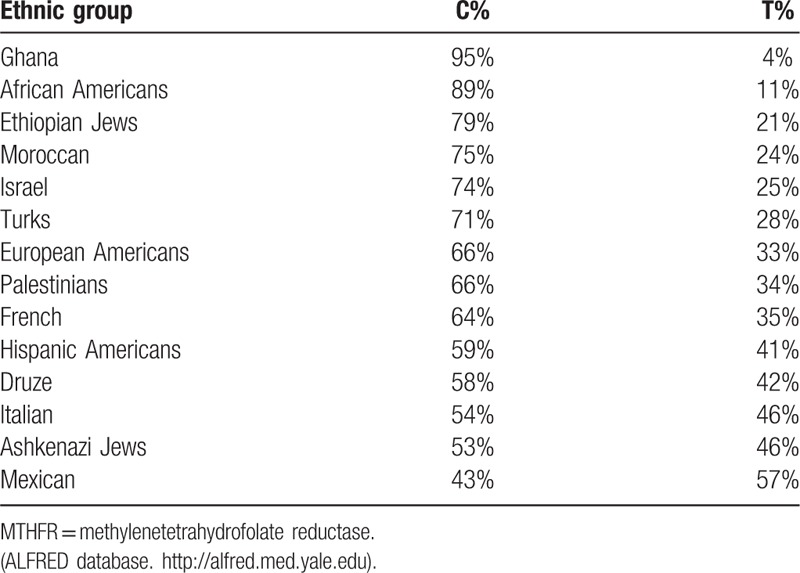
MTHFR C677T allele frequencies among different populations.

In addition, the risk effect may dependent on gene methylation, and thus, the gene–environment interaction between the genotypes and dietary intake. Folic acid consumption, in particular, is essential to maintain, or alter, the effect of the polymorphic variants.^[32]^

Our results show that the MTHFR C677T polymorphism is a relevant genetic risk factor for IBD in certain population. It seems that in the future we will have to take into consideration the ethnicity in order to find the specific genetic polymorphisms that are relevant to IBD.
